# Effects of a brief mindfulness-based intervention on emotional regulation and levels of mindfulness in senior students

**DOI:** 10.1186/s41155-018-0099-7

**Published:** 2018-08-02

**Authors:** Roberto Chiodelli, Luana T. N. Mello, Saul N. Jesus, Ilana Andretta

**Affiliations:** 1 Universidade do Vale do Rio do Sinos–Unisinos, Av. Unisinos, n° 950, Bairro Cristo Rei, São Leopoldo, RS 93022-750 Brazil; 2 Universidade do Algarve, Faro, Algarve Portugal

**Keywords:** Brief mindfulness-based intervention, Senior students, Undergraduates, Emotion regulation, Amount of meditative practices

## Abstract

Mindfulness-based interventions have been applied in diverse populations and achieved mental health benefits. This study examined the effects of a brief mindfulness program for emotional regulation and levels of mindfulness on senior students in Brazil. The intervention consisted of six weekly meetings attended by 30 participants. It is a pre-experimental research, with pre- and post-test comparative and correlation measurements. The preliminary results, which relied on parametrical and non-parametrical tests, revealed a reduction in total emotional regulation difficulties (*p* = 0.0001; *r* = − 0.55). Also, there was an increase in the levels of mindfulness in the subtests for both dimensions under evaluation: “Awareness” (*p* = 0.0001; *d* = 0.77) and “Acceptance” (*p* = 0.048; *d* = 0.37). By associating the amount of meditative practices performed by students with the variables, a significant positive correlation was found with the mindfulness dimension “Awareness” (*r*_P_ = 0.422; *p* = 0.020), and there was a significant negative correlation with Difficulties in emotion regulation (*r*_S_ = − 0.478; *p* = 0.008) and with its respective subscales “Non-acceptance” (*r*_S_ = − 0.654; *p* = 0.0001) and “Clarity” (*r*_S_ = − 0.463; *p* = 0.010). In conclusion, the application of a brief mindfulness-based intervention is promising in Brazilian university contexts; moreover, it can bring benefits to students, e.g., an increase in emotion regulation as well as in levels of mindfulness. We suggest that further research should use an experimental design and follow-up.

## Background

Mindfulness-focused practices propose a way for individuals to relate to their thoughts and emotions whereby they are led to understand experiences with acceptance and compassion rather than avoidance, control, or suppression (Hayes & Feldman, 2004; Roemer, Williston, & Rollins, 2015). Mindfulness is contrasted with mental states, in which attention is focused elsewhere (e.g., concerns, memories, fantasies, or plans), and with automatic behaviors, which lack awareness of our present-moment actions (Brown & Ryan, 2003). Mindfulness-based interventions (MBIs) stimulate cognitive defusion, in which individuals observe their thoughts without assuming that they are true or important. Thus, there is a reduction of the behavioral impact over thoughts, i.e., of reactivity relative to internal experiences (Baer, 2006).

Cardaciotto, Herbert, Forman, Moitra, and Farrow (2008) conceptualize mindfulness as our tendency to be highly aware of our inner and outer experiences with a posture of acceptance rather than one of judgment as to these experiences. Therefore, there are two fundamental and independent mindfulness dimensions: Acceptance and Awareness. Awareness refers to the monitoring of internal or external experience at the moment it occurs, in such a way that we do not exhibit mechanical behavior. The acceptance construct refers to an attitude of openness and non-judgment, free of defenses, beliefs and interpretations of one’s internal or external experience (Cardaciotto et al., 2008), which may also be related to the tendency of noticing the experience in a complete manner, without avoidance.

In 1979, Professor Jon Kabat-Zinn developed the first MBI, named mindfulness-based stress reduction (MBSR) (Kabat-Zinn, 2003; Kabat-Zinn et al., 1992). Although he practiced Buddhist meditation, he removed the religious framework and introduced MBSR into a scientific context. Other MBIs were created from MBSR. According to Chiesa and Malinowski (2011), the most widely used and recognized MBIs are the following: (a) mindfulness-based cognitive therapy (MBCT) (Segal, Williams, & Teasdale, 2002; Williams, Russell, & Russell, 2008), (b) mindfulness-based dialectical behavior therapy (DBT) (May, Richardi, & Barth, 2016; Shearin & Linehan, 1993), and (c) mindfulness-based acceptance and commitment (ACT) therapy (Robinson, Wicksell, & Olsson, 2005). Such programs are receiving increasing attention in modalities of potential treatment for a variety of psychosocial problems (Baer, 2006). These problems include chronic pain (Kabat-Zinn, Lipworth, & Burney, 1987; Zeidan, Gordon, Merchant, & Goolkasian, 2010), relief of physical symptoms associated with stress (Smith et al., 2011), depression (Shapero et al., 2008), and anxiety (Kabat-Zinn et al., 1992).

There has been growing interest in MBIs in recent years, and many studies have been conducted in both clinical and non-clinical populations. In one of these studies, Ruocco and Direkoglu (2013) evaluated the association of neurocognitive indices of attention and working memory with mindfulness in 55 neurologically and psychiatrically healthy adults. Attention and working memory tests and a two-dimensional mindfulness scale (the Philadelphia Mindfulness Scale) were completed. It was found that “Awareness” was associated with a variability measure of sustained attention response speed (SART), while “Acceptance” was more strongly connected with working memory efficiency. Also in an academic setting, Erogul, Singer, McIntyre, and Stefanov (2014) evaluated whether an abridged MBI intervention, adapted from MBSR, could improve wellness measures in first-year students at a medical school in New York. Their sample consisted of 58 participants who were randomly divided between experimental and control groups. The experimental group achieved a significant increase in self-compassion scores at the conclusion of the study and 6 months later. There was a significant reduction in perceived stress scores after the program had been completed, but not after a 6-month follow-up. The conclusion was that a brief MBSR intervention improves perceived stress and self-compassion in first-year medical students and can be a valuable instrument to improve their well-being and professional development.

The university context places considerable demands on students, e.g., examinations, internships, and class assignments. In Brazil, most university students have a professional activity. According to a survey conducted by the Institute of Applied Economic Research (IPEA, 2012), 58.3% of Brazilian undergraduates have a part-time or full-time employment. Milsted, Amorim, and Santos (2009) evaluated incidence of stress among psychology students at two private educational institutions in Curitiba. There were 117 students ranging in age from 18 to 48; 57 of them both went to college and had a job while 60 only went to college. Their findings showed that 71.79% of the students felt stressed, which is indicative of high incidence of stress among college students; they also found that studying and working may increase vulnerability to stress.

In Brazil, throughout the senior academic year of graduation, undergraduates must write their undergraduate thesis. Furthermore, it is a turning point, as students are expected to transition between roles: from students to workers (Bardagi & Boff, 2010). This transition from the role of a student to the role of a professional is usually critical for undergraduates and may increase their anxiety and stress. If students are unable to manage such sensations properly, the latter can interfere with learning and may even cause students to fail exams (Gama, Moura, Araújo, & Teixeira-Silva, 2008).

Other consequences of high stress levels in the academic setting (attention and concentration deficits, memorization and problem-solving difficulties, low productivity, and procrastination) might be a failure of emotional self-regulation to the extent that we fail to act as intended (Tourinho, 2008). Gratz and Roemer (2004) argue that the concept of emotional regulation involves (a) acceptance of emotions, (b) awareness and understanding of emotions, (c) ability to control impulsive behaviors and behave according to desired goals when negative emotions are experienced, and (d) ability to use appropriate emotional adjustment strategies in order to meet individual goals and situational demands. The relative absence of any of these abilities may indicate the presence of difficulties in emotional regulation. Besides training attention, MBIs develop the skill of maintaining an open and accepting attitude toward experience, which may be important for emotion regulation and affective outcomes (Slutsky, Rahl, Lindsay, & Creswell, 2016). Some studies have reported the influence of the construct “Acceptance” on emotional regulation. Teper and Inzlicht (2014) indicated that experienced meditators—who had higher mindfulness levels—made fewer errors in the experimental task, which mediated executive control. They concluded that participants with greater capacity to accept the impact of making a mistake can face the affective state more quickly. Consequently, they are more likely to observe their mistakes and prevent them from happening in future tests. This research also revealed that experienced meditators have greater evoked potentials generated in the anterior cingulate cortex, which is a brain area associated with executive functions (Teper & Inzlicht, 2014).

MBIs recommend that participants should add meditative practices to their daily routine during the course of the program. Subjects’ engagement with such interventions predicts that better results will be achieved (Creswell, 2017; Shapiro, Oman, Thoresen, Plante, & Flinders, 2008). Carmody and Baer (2008) investigated the relationship between practice time of suggested exercises and levels of mindfulness, medical and psychological symptoms, perceived stress, and psychological well-being in 174 adults in an MBSR clinical program. The time spent practicing formal meditation exercises has been significantly associated with the extent of improvement in most facets of mindfulness and various measures of symptoms and well-being. In a controlled study by Crane et al. (2014), 99 randomized participants with a history of at least three episodes of major depression engaged in an MBCT and completed assessments of their practice during 7 weeks of treatment. Recurrence of major depression was assessed immediately after treatment and at 3, 6, 9, and 12 months post-treatment. The results showed that participants who reported having practiced at home for at least 3 days a week during the treatment were half as likely to relapse compared to others.

Clearly, more research is needed to evaluate the effects of MBIs in order to gain more insights about their possible effects, especially in Brazil, since studies in this field are still scarce. A larger number of studies may allow the replication of such programs in different social contexts, thus causing a significant impact on public health. The objective of the present study is to identify possible effects of a brief MBI on emotional regulation and levels of mindfulness in university students. In addition, we seek to evaluate the association between the number of practices performed and the results achieved.

## Methods

### Design

The present study used a pre-experimental design, with pre- and post-test comparative and correlational measurements (Creswell, 2007).

### Participants

Thirty senior students regularly enrolled in different undergraduate courses at a university in Rio Grande do Sul (Brazil) participated in this study. As far as size is concerned, this sample has the minimum quantity required in pre-experimental quantitative studies, i.e., 15 participants, according to Sampieri, Collado, and Lucio (2013). Table 1 shows data on gender, age, marital status, and number of university students working during the intervention. The students were pursuing academic degrees in a variety of 15 undergraduate courses, including Administration (*n* = 12, 40%), Law (*n* = 3; 10%), and Social Work (*n* = 3; 10%). The most approximate family income was “above four minimum wages” (*n* = 15; 50%), followed by “between two and three minimum wages” (*n* = 6; 20%).

**Table 1:** Socio-demographic data (*n* = 30)

Characteristics	Values
Age	29.63 (SD = 8.38)
Gender	
Female	23 (76.66%)
Male	7 (23.33%)
Working	
Yes	23 (76.66%)
No	7 (23.33%)
Marital status	
Married	12 (40.00%)
Single	18 (60.00%)

As inclusion criteria, participants should attend at least four intervention sessions (66.66% attendance), be regularly enrolled in a university undergraduate program in the period of their undergraduate thesis, and have responded to all pre-test (T1) and post-test (T2) instruments.

### Instruments

The following data collection instruments were used in the present study:

- The Sociodemographic Questionnaire—designed to collect data such as age, sex, income, marital status, course, and employment.
- The Philadelphia Mindfulness Scale (PHLMS)—developed by Cardaciotto et al. (2008) and translated and adapted to the Brazilian Portuguese by Silveira, Castro, and Gomes (2012). It consists of a 5-point Likert scale and 20 items, divided in two dimensions: “Acceptance” and “Awareness.” Both dimensions presented internal consistency of 0.85 and 0.81, respectively. In the present study, Cronbach’s alpha in T1 was 0.75 for “Acceptance” and 0.75 for the “Awareness” dimension, while in T2, Cronbach’s alpha was 0.87 for the “Acceptance” dimension and 0.78 for “Awareness.”
- Difficulties in Emotion Regulation Scale (DERS-36)—developed by Gratz and Roemer (2004) and translated and adapted to European Portuguese by Coutinho, Ribeiro, Ferreirinha, and Dias (2010). It assesses the following typical levels of emotional deregulation in six domains, quoted with their respective Cronbach alphas: “Non-acceptance”—non-acceptance of emotional responses (0.86), “Goals”—difficulties engaging in goal-directed behavior (0.85), “Impulse”—impulse control difficulties (0.80), “Awareness”—lack of emotional awareness (0.74), “Strategies”—limited access to emotion regulation strategies (0.88), and “Clarity”—lack of emotional clarity (0.75). It contains 36 items on a 5-point Likert scale ranging from 1 (almost never) to 5 (almost always). The scale indicated high values of internal consistency (0.93) in the study by Coutinho et al. (2010). Portuguese adaptation of the Brazilian reality was used. In the present study, total DERS presented Cronbach alpha values of 0.89 in T1 and 0.88 in T2. The Cronbach alphas for the subscales were as follows: “Non-acceptance” (T1 = 0.82; T2 = 0.86), “Goals” (T1 = 0.81, T2 = 0.86), “Impulse” (T1 = 0.80, T2 = 0.75), “Awareness” (T1 = 0.72, T2 = 0.81), “Strategies” (T1 = 0.73, T2 = 0.80), and “Clarity” (T1 = 0.82, T2 = 0.55).
- Self-report form of meditative practices—prepared by the author. It served to record the number of suggested practices that participants performed in the interval of each meeting, as well as participants’ attendance.

### Procedures

The application of a brief MBI in senior students was inserted in an extension course called “TCCendo sem estresse” (“Writing my undergraduate thesis less stressfully”), which is organized by the University’s Academic Service Support Unit (UASS). The UASS issued the authorization letter to the program. The aim of “TCCendo sem estresse” is to provide senior students with technical and psychological support while they are writing their undergraduate thesis. The University’s Research Ethics Committee approved the project (CEP No. 15/251) in accordance with the provisions of the National Health Council Resolution 466/12.

A pilot project was applied at the beginning of the second academic semester of 2015, and there was a positive response from the students in a satisfaction survey conducted by the UASS. Based on this experience, the program was enhanced and applied (groups 1 and 2) of “TCCendo sem estresse” at the beginning of the first academic semester—from March to May of 2016. The same program was conducted (group 3) at the beginning of the second academic semester—from August to October of 2016.

**Fig. 1:**
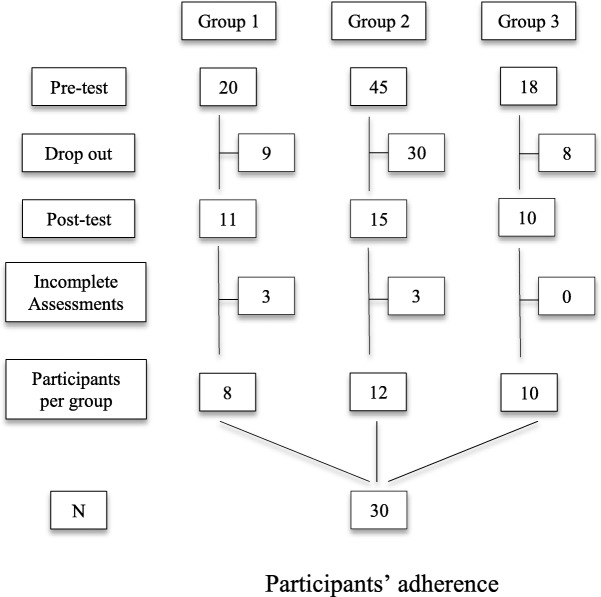
Participants’ adherence

The students were invited by email to attend the course and there were 20 vacancies in each group. Because of an oversight in group 2’s entries, 45 people were accepted on the first day. Figure 1 shows the adherence of senior students in the three groups.

By analyzing Fig. 1, it is noticed that there was a high number of dropouts. The fact that senior students’ participation was free of any charge and they had no academic obligation to participate in the course may have corroborated to the phenomenon. However, participants’ attendance actually increased considerably in relation to the “TCCendo sem estresse” course groups that did not contain the brief MBI, as shown on Table 2.

**Table 2:** Final adherences between courses with and without brief MBI

Final adherence	Course with brief MBI	Course without brief MBI
Group 1	8	3
Group 2	12	0
Group 3	10	1

*Note*: Both courses occurred in the same period and with different participants

### Intervention

This brief MBI was adapted from the book “Mindfulness: How to Find Peace in a Frantic World” by Williams and Penman (2015, see Table 3). This book is based on mindfulness-based cognitive therapy (MBCT). The intervention was adapted according to the timetable of the course “TCCendo sem estresse”. It consisted of six meetings; the first and the last meetings lasted for 1 h 15 min while the remaining ones lasted for 35 min. In the first meeting, the senior students who agreed to participate in the study received information about the program and signed the Free and Informed Consent Form. Also in the first meeting, students answered the following instruments: DERS-36, PHLMS, and the socio-demographic questionnaire. DERS-36 and PHLMS were applied again in the sixth and final meeting. In each meeting, participants filled a self-report form of meditative practices, where they recorded the number of practices they had executed since the last meeting they attended.

**Table 3:** Brief mindfulness (Mfs) program—“TCCendo sem estresse”

Description	Activities
Week 1 Introduction	- Presentation; - Ice-breakers; - What is Mfs; - Self-compassion; - Body activation + body scan practice; - Daily task: 10-min guided daily meditation (track 1).
Week 2 Mindfulness and the body	- Group sharing on the previous week; - Body awareness; - Activity: mindful movements + mindful eating; - Daily task: guided meditation (track 3) and then 3 min in silence, concentrating on the breath.
Week 3 Mindfulness and emotions	- Group sharing on the previous week; - Emotions × thoughts; - Acceptance; video activity: observe one’s body reaction; - Daily task: guided meditation (track 6). Then, remain silent for 2 min, focusing on sounds.
Week 4 Mindfulness on a daily basis	- Group sharing on the previous week; - Activity: mindful walking; - Daily task: choose between tracks 1, 3, or 6. Then practice Mfs during a specific chosen activity (tooth brushing; eating; cooking; washing the dishes; driving, etc.).
Week 5 Mindfulness and the final paper	- Group sharing over the past week; - Procrastination and Mfs; - Activity: effects of specific words on bodily sensations; - Daily task: guided meditation number 5.
Week 6 “Week 6 is the rest of your lives”	- Group sharing on the previous week and the whole program; - Activity: body scan; - Delivery of material with suggestions on how to cultivate a mindful living.

*Note.* Adapted from: Williams and Penman (2015). Guided meditations are from the CD of the same book

In addition to psychoeducational and dynamic practices implemented in the meetings, participants received follow-up through group emails. The purpose of these emails was to engage participants in the proposed daily tasks, provide guided meditations audio, update students on the contents and activities of the previous meeting (specially those who had missed the meeting), and provide any kind of support needed.

The intervention was coordinated by the researcher, who has 10 years of meditation experience, with participation in programs and courses about mindfulness. The meetings were observed by a psychologist or a social worker, both professionals from the UASS. Each meeting addressed a topic: (1) project presentation and the concept of mindfulness, (2) the body and mindfulness, (3) mindfulness and emotions, (4) mindfulness on a daily basis, (5) mindfulness and the undergraduate thesis, and (6) week 6 is the rest of your lives.

### Data analysis

Initially, descriptive statistics was performed through averages, medians, standard deviations, and frequencies in order to characterize the sample. Then, because the sample is below 50, the Shapiro-Wilk test was applied in order to evaluate symmetry of data distribution of each test and subtest. The variables of DERS-36 did not present an approximately normal distribution (*p* > 0.05). For these variables, which were considered as non-parametric, the Wilcoxon signed-rank test was used in order to compare the pre- and post-intervention evaluations, while the paired Student’s *t* test was applied for the variables with a normal distribution (PHLMS “Acceptance” and PHLMS “Awareness”). The bootstrap confidence interval was generated on the comparative tests, which estimates the empirical amplitude for the minimum levels of significance.

Pre- and post- comparisons were reported on the total number of cases (*n* = 30). It was not possible to compare the three groups (ANOVA technique), due to the fact that neither the normality of data distribution nor the homogeneity of variances occurred. Besides that, the groups had non-representative sample sizes. These characteristics may overestimate data independence and indicate that the *p* value might contain a significant error (Callegari-Jacques, 2003; Singer, Poleto, & Rosa, 2004). Considering that, effect sizes should be a more reliable source of information.

Effect size was calculated using Cohen’s *d* for the dimensions analyzed by the paired *t* test with the formula *d* = *t*/√*n*, while Pearson’s *r* (Pallant, 2013) was used for the dimensions calculated with Wilcoxon signed-rank test. The formula in use was *r* = *Z*/√2*n*. It should be mentioned that the categorization of effect size for Cohen’s *d* is small up to 0.50, medium to 0.80, and large from 0.80. In contrast, the effect size levels for Pearson’s *r* are small up to 0.10, medium to 0.30, and large from 0.50.

In order to correlate the difference between the results and the total number of meditative practices, Pearson’s correlation was measured for the parametric dimensions (PHLMS) while Spearman’s correlation was calculated for the non-parametric dimensions (DERS-36). In addition, the degree of reliability or internal consistency (Cronbach’s alpha) of each variable was evaluated, as well as confidence intervals (95%). All analyses were performed using the Statistical Package Social Sciences software (SPSS version 22, IBM, 2012), and statistical significance was established at the level of 5%.

## Results

Table 4 shows the effects relative to the pre- and post-test of the brief MBI on senior students. The analysis of the data revealed a significant effect in all variables evaluated. There was an increase in the “Awareness” PHLMS dimension (*p* = 0.0001), with a medium effect size (*d* = 0.77). The students also showed a rise in PHLMS “Acceptance” (*p* = 0.048), with a small effect size (*d* = 0.37). The participants showed a reduction in Difficulties in emotion regulation (DERS-36) (*p* = 0.0001), with a large effect size (*r* = − 0.55). There were also decreases in all the subscales: “Non-acceptance” (*p* = 0.005, *r* = − 0.38), “Goals” (*p* = 0.018, *r* = − 0.31), Impulse (*p* = 0.001, *r* = − 0.44), Awareness (*p* = 0.003, *r* = − 0.39), and “Clarity” (*p* = 0.005; *r* = − 0.36), with a medium effect size. The “Strategies” subscale (*p* = 0.0001; *r* = − 0.50) presented a large effect size.

**Table 4:** Pre- and post-test effects of a brief mindfulness-based intervention on senior students (*n* = 30)

Instrument and evaluation		Mean	Median	SD	*t*/*Z*	*p*	CI *p I*	*d*/*r*
PHLMS_Awareness^1^	Pre	24.67	24.50	5.39	4.23	0.0001***	0.000011–0.00036	0.77^aa^
	Post	28.83	29.00	5.79				
PHLMS_Acceptance^1^	Pre	17.40	19.00	5.78	2.06	0.048*	0.02581–0.0681	0.37
	Post	19.93	20.00	7.15				
DERS_TOTAL^2^	Pre	84.90	85.00	17.05	− 4.26	0.0001***	0.0000–0.00046	− 0.55^aaa^
	Post	70.23	66.00	13.33				
DERS_non-acceptance^2^	Pre	13.37	12.50	5.38	− 2.98	0.003**	0.00206–0.00514	− 0.38^aa^
	Post	10.80	10.50	4.08				
DERS_goals^2^	Pre	15.87	17.00	4.03	− 2.36	0.018*	0.01476–0.02164	− 0.31^aa^
	Post	13.83	13.50	3.82				
DERS_impulse^2^	Pre	12.37	12.00	4.06	− 3.47	0.001**	0.00000–0.00092	− 0.44^aa^
	Post	9.80	9.00	3.07				
DERS_awareness^2^	Pre	15.90	15.50	4.35	− 3.02	0.003**	0.00044–0.00236	− 0.39^aa^
	Post	13.50	13.50	4.07				
DERS_strategies^2^	Pre	16.67	17.00	4.63	− 3.91	0.0001***	0.00000–0.00046	− 0.50^aaa^
	Post	13.47	12.00	3.73				
DERS_clarity^2^	Pre	10.73	10.50	3.73	− 2.79	0.005**	0.00019–0.00181	− 0.36^aa^
	Post	8.83	8.50	2.11				

*Note*. ^1^Variables with normal distribution analyzed by the paired *t* test; effect size: *d*. ^2^Non-parametric variables analyzed by the Wilcoxon signed-rank test (*Z*); effect size: *r*. *p < 0.05; **p < 0.01; ***p < 0.001. ^aa^Medium effect size. ^aaa^Large effect size. Confidence interval was 95% for the test level of significance

Table 5 shows the correlation between number of meditative practices and the variables evaluated. The mean number of practices performed per participant was 14.70 (SD = 10.01). The number of practices ranged from zero to 38. Among the nine variables analyzed, four presented a significant correlation with number of practices. There was a positive correlation with the “Awareness” mindfulness dimension (*r*_P_ = 0.422; *p* = 0.020), and a negative correlation with Difficulties in emotion regulation (*r*_S_ = − 0.478, *p* = 0.008), as well as in the “Non-acceptance” (*r*_S_ = − 0.654, *p* = 0.0001) and “Clarity” (*r*_S_ = − 0.463; *p* = 0.010) subscales.

**Table 5:** Correlation between each variable and number of meditative practices performed by senior students (*n* = 30)

Instruments and evaluations	Amount of meditative practices	
	*r*_P_/*r*_S_	*p*
PHLMS Awareness^1^	0.422	0.020*
PHLMS Acceptance^1^	0.162	0.393
DERS Total^2^	− 0.478	0.008**
DERS Non-acceptance^2^	− 0.654	0.0001***
DERS Goals^2^	− 0.346	0.061
DERS Impulse^2^	− 0.054	0.777
DERS Awareness^2^	− 0.314	0.091
DERS Strategies^2^	− 0.112	0.555
DERS Clarity^2^	− 0.463	0.010*

*Note*. ^1^Variables with normal distribution analyzed by Pearson’s correlation (*r*_P_). ^2^Non-parametric variables analyzed by Spearman’s correlation (*r*_S_); *p < 0.05; **p < 0.01; ***p < 0.001

## Discussion

The intervention proved to achieve the proposed objectives satisfactorily and led to significant changes in all variables analyzed. It is important to note that this study relies on a small sample size and does not utilize a control group comparison. Therefore, the results are seen as preliminary and informative, insofar, they reflect an MBI feasibility in a Brazilian university setting.

There was a medium effect size increase of the “Awareness” mindfulness dimension in senior students, which indicates that the program developed a greater continuous monitoring of the experience. Students’ ability to recognize what they feel or think gives them more autonomy for decision-making. The Awareness construct (Cardaciotto et al., 2008) implies that the individual is aware of the inner and outer experience as a whole but not limited to anything specific. It differs from Attention, which can be defined as greater sensitivity to a narrow range of experiences (Kosslyn & Rosenberg, 2001) and implies that out-of-focus experience is actively ignored or disregarded.

There was a small effect size increase of the “Acceptance” dimension, which sustains that the program supported the development of present-day consciousness in a nonjudgmental, compassionate, and open posture to the experience. This construct has been defined as “experiencing events fully and without defense as they are” (Hayes, 1994, p. 32). During “Acceptance,” one is open to the reality of the present moment without being in a state of belief or disbelief (Roemer & Orsillo, 2003). Cardaciotto et al. (2008) argue that acceptance in this context should not be confused with passivity or resignation. Rather, it means being present, as a substitute for avoiding or worrying about events as they occur.

The significant decrease of difficulties in emotion regulation with a large effect size suggests that the intervention has been productive in this regard. The DERS-36 measure proposes an integrative conception of emotional regulation, involving not only emotional arousal modulation, but also awareness, understanding, and acceptance of emotions, as well as the ability to act as desired regardless of emotional state (Gratz & Roemer, 2004). By developing the ability to deal with emotions in a consistent manner, senior students can better manage the pressure and the demands of a critical moment of their lives, which involves completion of their undergraduate thesis and the transition from the academic to the professional environment.

The relationship between mindfulness and emotion regulation was addressed in several studies. Grice (2015), for instance, sought to better understand psychological constructs as possible risk factors for young female students in the development of anorexia nervosa. This study examined 119 undergraduate students without prior diagnosis of eating disorder in order to explore the relationship between mindfulness, emotion regulation, and eating disorder symptoms in a nonclinical environment. The participants completed a cross-sectional survey that included the Five Facet Mindfulness Questionnaire (FFMQ)-short form, the Difficulties in Emotion Regulation Scale (DERS-36), and the Eating Disorder Inventory (EDI-3). The results indicated that individuals with more typical eating disorder symptoms had lower levels of mindfulness and greater difficulties in regulating their emotions. The relationship between mindfulness and eating disorders was identified as mediated by emotion regulation.

Goldin and Gross (2010) examined MBI-related changes in the brain-behavior indices of emotional reactivity and regulation of negative self-beliefs in patients with social anxiety disorder (SAD). Fourteen patients underwent functional magnetic resonance imaging while reacting to negative self-beliefs and regulating negative emotions. Compared with baseline, the patients who completed the MBSR program showed improvement in anxiety and depression symptoms and self-esteem. The participants showed (a) decreased negative emotion experience, (b) decreased amygdala activity, and (c) increased activity in brain regions involved in attention deployment during the breath-focused attention task. It was concluded that MBSR in patients with SAD can reduce emotional reactivity and increase emotional regulation.

In the present study, all DERS-36 subscales obtained a significant decline and consistent effect size. The “Strategies” factor, which presented a large effect size, is particularly worth of notice. Such a construct means limited access to emotional regulation strategies and reflects a belief that little that can be done to regulate emotions effectively when an individual is upset. Therefore, it is understood that students have increased their range of strategies to deal with negative emotions.

The “Non-Acceptance” factor reflects a tendency to show negative secondary emotional responses to negative emotions, such as guilt, shame, anger, or judgment as weak when the individual does not feel well. These results indicate that there was a medium effect size decrease in secondary negative responses to negative emotions, thus generating less internal conflict and reducing the distance from the perceived emotion. There is a notable difference between the constructs “Non-Acceptance” of DERS-36 and “Acceptance” of PHLMS. They are distinct, as the former addresses a negative reaction to an initial negative emotion, while the “non-acceptance” of the latter is focused on the act of wishing to avoid a feeling or a thought through distractions.

The “Goals” factor reflects difficulties of concentration and accomplishment of tasks when experiencing negative emotions. It is closely related to the concept of procrastination. In the educational context, procrastination manifests itself through the action of postponing the commitment to study or complete tasks requested by teachers (Costa, 2007). The delayed task is replaced with another less important (and often more pleasurable) one; however, the individual who acts this way feels uncomfortable with the situation (Schouwenburg, 2004). In this regard, the results suggest that the senior students increased their capacity of focus and action for performing the necessary tasks with a medium effect size.

The “Awareness” factor reflects lack of attention and awareness of one’s own emotional responses. In this study, the senior students presented a higher ability to recognize their emotional responses, with a medium effect size. It can be seen that emotional awareness fostered impulsivity reduction. The “Impulse” factor refers to the individual’s difficulties in remaining in control of his or her behavior by experiencing negative emotions. Therefore, the undergraduates participating in this research developed lower reactivity to stressful events, presenting a medium effect size. Finally, the “Clarity” factor denotes lack of emotional clarity and shows the extent to which individuals know and are aware about the emotions they are experiencing. It indicates that students increased their emotional clarity, with a medium effect size, after the program. It should be mentioned that the constructs Awareness and Clarity from DERS have a strong relationship with PHLMS “Awareness.”

The correlation between number of practices performed and the variables PHLMS “Awareness,” “DERS_Total,” “Non-Acceptance,” and “Clarity” supports the idea that meditative practices are compared to an exercise that should be practiced regularly. These findings corroborate the findings of Hawley et al. (2014) and Perich, Manicavasagar, Mitchell, and Ball (2013), who evaluated the same association and concluded that participants who performed more mindfulness practices showed a more consistent improvement in mental health compared to participants who practiced fewer practices. However, there is still no well-established association between the amount of time an individual engages in mindfulness practices and the subsequent relief of symptoms. An extensive systematic review (Vettese, Toneatto, Stea, Nguyen, & Wang, 2009) evaluated the relative impact of mindfulness practice on MBCTs and MBSRs. Among the findings, only eight of the 24 studies reviewed by the authors showed evidence for the relationship between mindfulness practice and clinical outcome. Although some hypotheses have been formulated, more specific research is necessary to better understand how mindfulness practices can influence desired outcomes.

This research applied a brief MBI (with fewer meetings and shorter duration per meeting). However, it produced similar outcomes (significant improvements) when compared to the traditional interventions, which are longer. Carmody and Baer (2009) reviewed the contact hour quantity of MBIs and their effect sizes for psychological distress. The correlation between the mean effect size and the number of hours in the classroom was not significant for both clinical and non-clinical populations; which suggests that MBI adaptations for populations that have less commitment time may be as efficient as traditional MBIs.

Even though this study was not designed to analyze an MBI feasibility in college environments, it is relevant to suggest some aspects and inform extra data for future research. Two points were assumed as decisive for a satisfactory brief MBI implementation: institution support (program propagation, staff assistance, proper classroom, and material provision) and the pilot program application (which prevents a number of issues and improves the quality of the actual research). In addition, a qualitative questionnaire was applied in the last brief MBI meeting in order to observe participants’ perception on their own commitment with the program and if they noticed any effects. The student’s commitment average, with a scale ranging from 0 to 5, was 3.53 (SD = 1.13). Twenty-nine out of 30 students reported the brief MBI had a positive effect in their lives. It is possible to infer the program was well accepted and successful considering participants’ satisfaction.

It is plausible to suggest that at the beginning of the semester students tend to have less anxiety and stress compared to the middle of the semester. The application of pre-tests occurred at the beginning of the semester (second or third week), while the post-test application was held in the eighth or ninth week, that is, in the middle of the semester, when students have more attributions in relation to the beginning of the semester. Although the moment of test application aspect was not statistically calculated, it may be considered positively noteworthy for the brief MBI.

## Conclusions

The present research has some important methodological limitations, such as a non-random assignment and no follow-up test. In addition, as seen previously, due to the small sample size of the parallel courses without the brief MBI, it was not possible to assess a control group. Another aspect that would give more reliability to the results is the application of non-self-reported instruments, such as the analysis of participants’ blood pressure or cortisol level.

Two interventions (groups 1 and 2) were conducted in the first academic semester of 2016, while the other intervention (group 3) was held in the second academic semester of the same year. It would be interesting to compare the three groups separately. However, as referred in the “Data analysis” section, a nested structure of the data could not be considered, implying an overestimation of data independence. Finally, the time of the meditative practices performed by each participant was not accurately evaluated. The proposed tasks, such as guided meditations, varied in time and were, on average, 10 min long.

Considering these limitations, the results are preliminary but encouraging for further studies in Brazilian university settings. The results infer that a brief mindfulness intervention in a university context can bring many benefits to senior students, e.g., increase their capacity for emotional regulation and their awareness of inner and outer experience. These attributes enable a greater ability to perform tasks, deal with one’s emotions, and, consequently, improve students’ mental health. The application of MBIs can be of great value to students’ academic achievement and psychosocial development. Further research is suggested for replication of the program, using a randomized controlled trial and follow-up.

## Abbreviations

ACT: Mindfulness-based acceptance and commitment therapy
DBT: Mindfulness-based dialectical behavior therapy
DERS-36: Difficulties in Emotion Regulation Scale
EDI-3: Eating Disorder Inventory
FFMQ: Five Facet Mindfulness Questionnaire
IPEA: Institute of Applied Economic Research
MBCT: Mindfulness-based cognitive therapy
MBIs: Mindfulness-based interventions
MBSR: Mindfulness-based stress reduction
PHLMS: The Philadelphia Mindfulness Scale
SAD: Social anxiety disorder
SART: Sustained attention response speed
SPSS: Statistical Package Social Sciences software
UASS: University’s Academic Service Support Unit
